# A novel bailout technique using myocardial biopsy forceps to grasp a dislodged angio-seal collagen with footplate

**DOI:** 10.1186/s42155-024-00487-x

**Published:** 2024-10-05

**Authors:** Hiromi Miwa, Naoki Hayakawa, Yasuyuki Tsuchida, Shinya Ichihara, Satoshi Hirano, Shunsuke Maruta, Kotaro Miyaji, Shunichi Kushida

**Affiliations:** Department of Cardiovascular Medicine, Asahi General Hospital, I-1326 Asahi, Chiba, 289-2511 Japan

**Keywords:** Angio-Seal, Endovascular therapy, Myocardial biopsy forceps, Vascular closure device, Iliac artery

## Abstract

**Background:**

Hemostatic devices are now frequently used in femoral artery punctures, and the Angio-Seal (Terumo, Tokyo, Japan) is one of the most commonly used devices for closure of the femoral artery because it provides rapid hemostasis. Although device failure rarely occurs, if the collagen falls into the femoral artery, it may lead to severe limb ischemia. Herein, we describe a case of a novel endovascular technique for the treatment of Angio-Seal arterial closure device failure.

**Case presentation:**

The patient in Case 1 was a 75-year-old man with severe left limb claudication. We used a contralateral antegrade approach and used the Angio-Seal for hemostasis. However, the Angio-Seal collagen and footplate dropped and stopped at the bifurcation of the superficial femoral artery and deep femoral artery. The collagen with the footplate was caught with myocardial biotome forceps (MBF) and pulled into the external iliac artery (EIA). The distal common femoral artery (CFA) was punctured, and we delivered a 10.0- × 80-mm stent (SMART^®^; Cordis, USA) to the EIA from the ipsilateral sheath. The stent was deployed at the EIA and crushed the collagen. The patient in Case 2 was an 88-year-old man with rest pain in the right limb. The right CFA was punctured using an ipsilateral approach and the Angio-Seal was used for hemostasis. The Angio-Seal collagen with the footplate dropped into the bifurcation of the deep femoral artery. The collagen and footplate were caught with MBF and pulled up to the EIA. The right CFA was punctured and a 10.0- × 60-mm stent (SMART^®^; Cordis) was delivered from the ipsilateral sheath. The stent was deployed at the EIA and crushed the collagen with the footplate.

**Conclusions:**

MBF were used to grasp the dislodged collagen with the anchor and cover it with a stent at the iliac artery. This may be a useful bailout technique for Angio-Seal dislodgement.

## Background

**Fig. 1:**
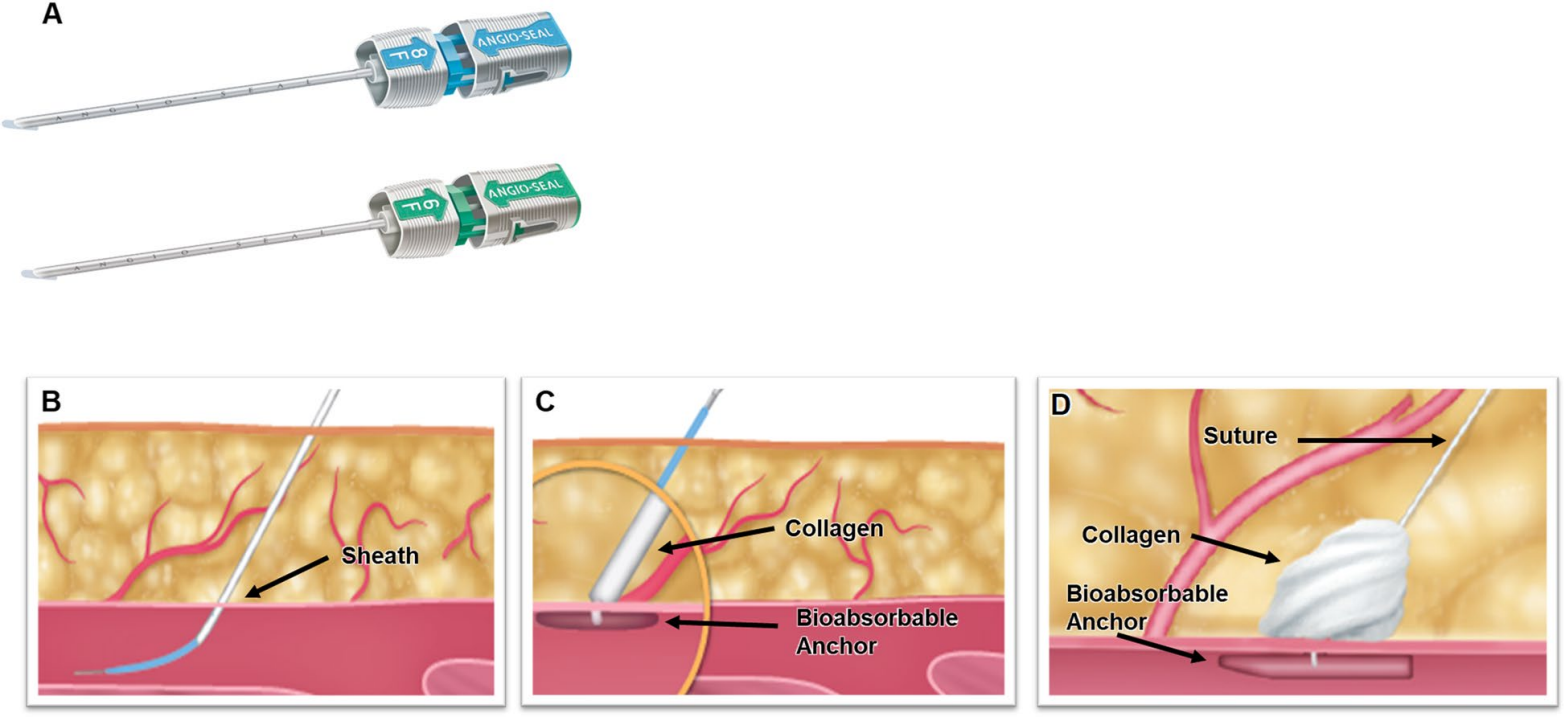
Structure of the Angio-Seal. **A** Outer sheath of Angio-Seal. **B/C** Schematic drawing of hemostasis with bioabsorbable anchor and collagen. **D** Schematic drawing showing the Angio-Seal device being used to seal a puncture site

 Hemostasis of femoral artery puncture sites is problematic in endovascular therapy (EVT) [1, 2]. Vascular closure devices are frequently used because they are safe and their usage leads to early mobilization and a shorter hospital stay (Brancheau et al. 2018; [3, 4]). The Angio-Seal (Terumo, Tokyo, Japan) is one of the most commonly used devices for hemostasis of the femoral artery and incorporates a collagen plug and a footplate to seal the puncture site (Fig. 1) [5].

The incidence of femoral artery occlusion due to failure of the Angio-Seal device is reported to be about 0.75% [5]. When femoral artery occlusion occurs, surgical treatment may be required to salvage the limb [6]. In the present report, we describe an effective, safe, and rapid endovascular bailout method for femoral artery occlusion after failure of the Angio-Seal hemostatic device using the MyoCardiaL biopsy forceps to grAsp a dislodged Angio-Seal collagen With footplate and CoveR it with A steNt at thE iliac artery (CLAW CRANE) technique.

## Case presentations

### Case 1

**Fig. 2:**
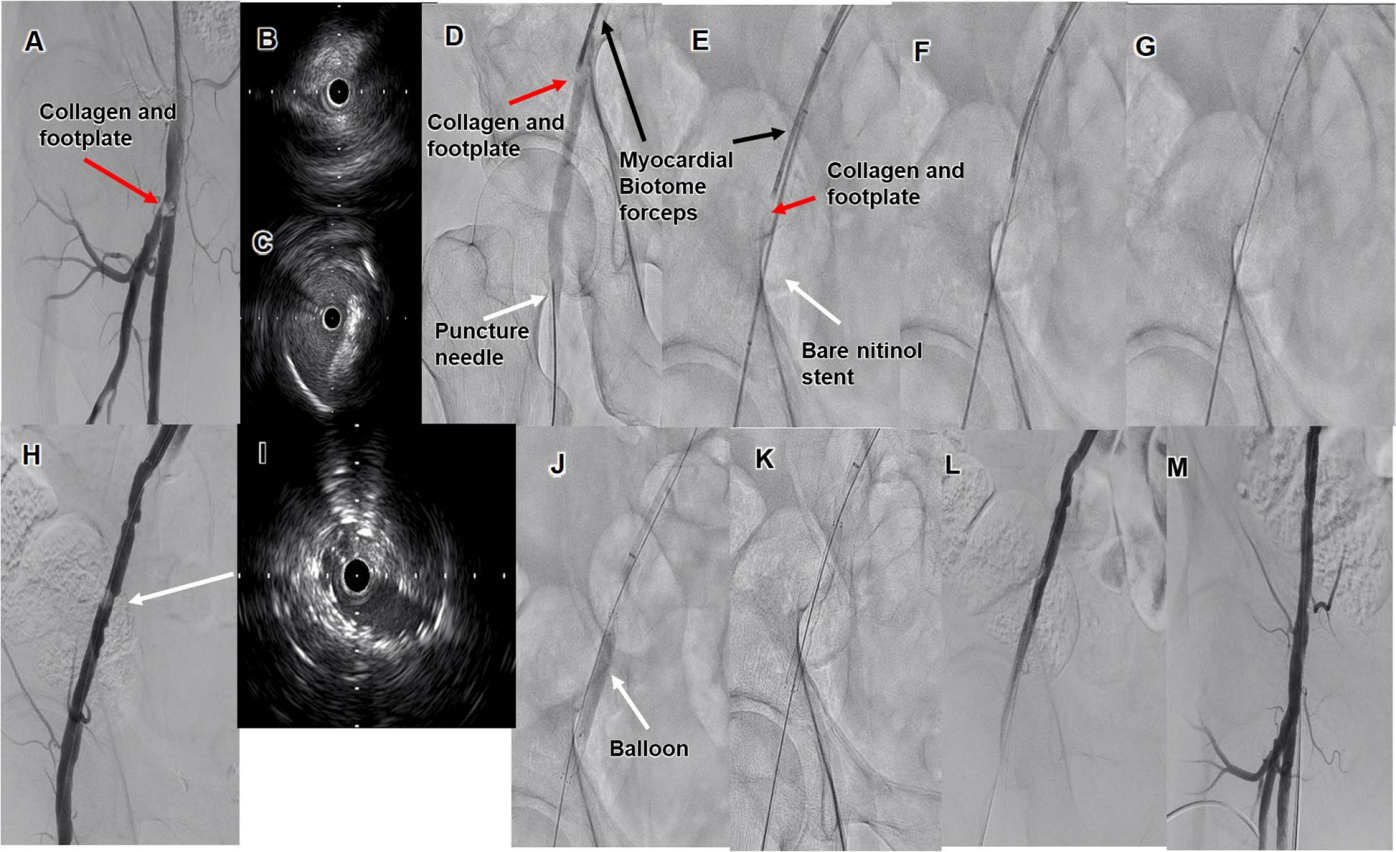
Angiogram of Case 1 after the Angio-Seal drop. **A** The Angio-Seal collagen stopped at the bifurcation of the deep femoral artery (DFA). **B** Intravascular ultrasound (IVUS) observation from the DFA shows that the Angio-Seal collagen occupies the DFA, and the DFA is almost occluded. **C** IVUS observation from the superficial femoral artery (SFA) shows that the Angio-Seal collagen is in the SFA but is not occluding the SFA. **D** A biotome grasps the Angio-Seal collagen and pulls it up to the external iliac artery (EIA). **E-F** While the biotome forceps grasp the collagen, a 10.0- × 80-mm stent (SMART^®^; Cordis, USA) is deployed to the EIA and the Angio-Seal collagen is positioned between the stent and the vessel wall. **G** After stent deployment, the stent expansion is not completed. **H** Angiography performed after stent deployment shows that the collagen-inserted stent portion is not fully dilated. **I** IVUS shows that the Angio-Seal collagen is pushing the stent. **J** Post-dilation of the stent. **K-L** Angiography performed after post-dilation shows that the stent is fully expanded

A 75-year-old man with hypertension, peripheral artery disease, and an old cerebral infarction presented with claudication of the left lower limb. The ankle–brachial index was 0.76 on the left side. A 6-Fr guiding sheath (Crossroads^®^ guiding sheath; NIPRO) was inserted into the right common femoral artery (CFA) after the angiography-guided femoral artery puncture. Control angiography showed a focal stenotic lesion in the left superficial femoral artery (SFA). We dilated a 5.0- × 150-mm drug-coated balloon (In.Pact Admiral^®^; Medtronic, Minneapolis, MN, USA). We used the Angio-Seal hemostatic device (Terumo) at the puncture site, but failed to achieve hemostasis. Duplex ultrasound (DUS) showed that the Angio-Seal collagen with the footplate had dropped and obstructed the femoral artery. A 6-Fr guiding sheath (Crossroads^®^ guiding sheath; NIPRO) was inserted into the left common CFA. Angiography showed that the Angio-Seal collagen with the footplate had stopped at the bifurcation of the SFA and deep femoral artery (DFA) (Fig. 2A). AnteOwl intravascular ultrasound (IVUS) (AnteOwl WR^®^ IVUS; Terumo) showed that the Angio-Seal collagen with the footplate had dropped into the DFA rather than the SFA and had almost occluded the DFA (Fig. 2B and C). Myocardial biotome forceps (MBF) were inserted and used to grasp the collagen and move it to the right EIA. We initially tried to retrieve the collagen straight into the guiding sheath, but the large size of the collagen prevented it from fitting inside the 6-Fr guiding sheath. Therefore, the distal CFA was punctured by angiography-guided and a 6-Fr short sheath was inserted (Fig. 2D). A 10.0- × 80-mm self-expandable bare nitinol stent (BNS) (SMART^®^; Cordis) was deployed to the EIA, so that the MBF grabbing the collagen were positioned between the BNS and the vessel wall (Fig. 2E and G). Angiography and IVUS showed that the stent was inadequately dilated (Fig. 2H and I). Therefore, the balloon was dilated and the stent was appropriately extended (Fig. 2J and K). The final angiography showed good antegrade flow without residual material (Fig. 2L and M). IVUS also confirmed that there was no residual collagen and footplate. Bilateral sheaths were hemostatic using Exoseal (Cordis, USA) hemostatic devices, and manual compression for 5 min was added when using the Exoseal hemostatic device. No additional puncture site-related complications were observed postoperatively. The time from the first angiogram to the last angiogram was 35 min. Aspirin and clopidogrel were administered postoperatively for 3 months, and then clopidogrel alone was continued.

### Case 2

**Fig. 3:**
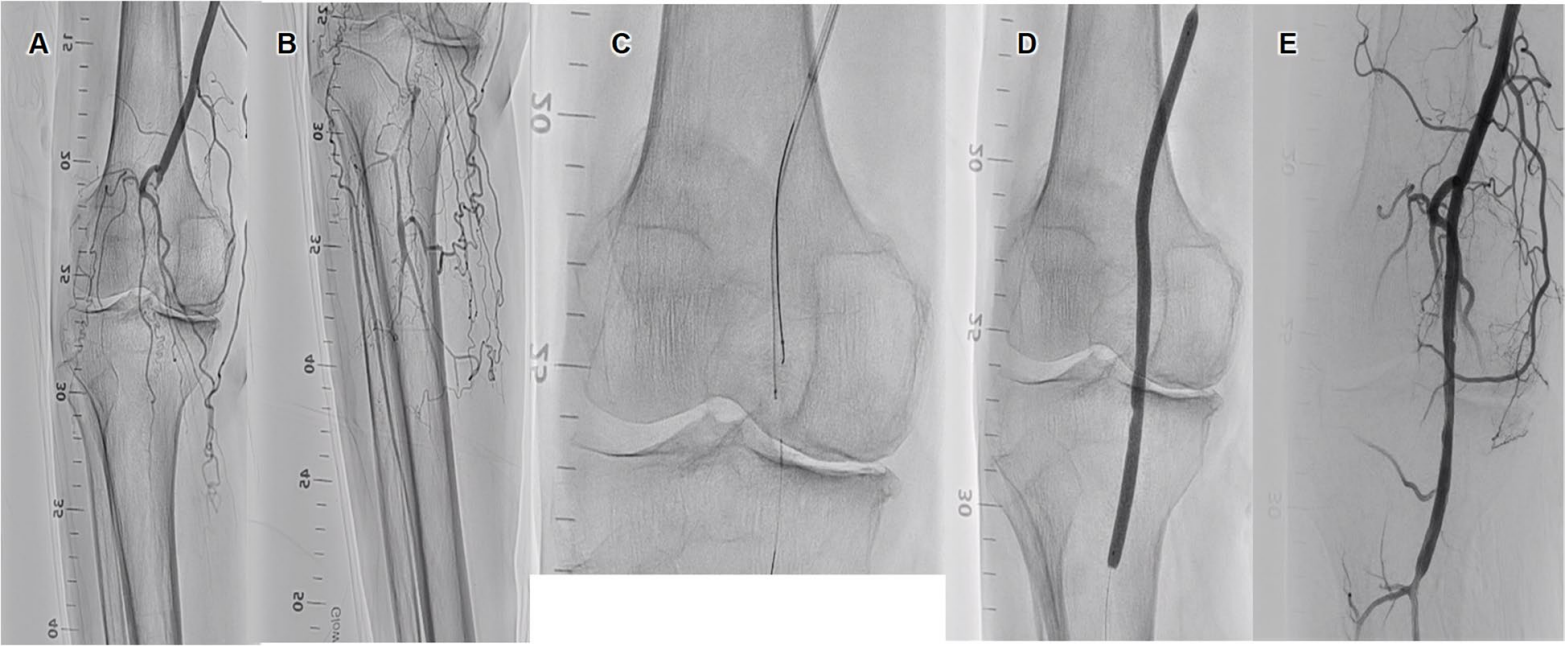
Angiogram of the endovascular treatment in Case 2. **A** Control angiography shows that the right popliteal artery is occluded from the proximal segment. **B** Vessels are contrasted from the distal portion through the collateral vessels. **C** Intravascular ultrasound-guided wiring is performed. **D** A 5.0- × 150-mm drug-coated balloon (Lutonix^®^; BD, USA) is dilated at the popliteal artery lesion to prevent restenosis. **E** The final angiography shows good antegrade flow with no dissection or residual stenosis

**Fig. 4:**
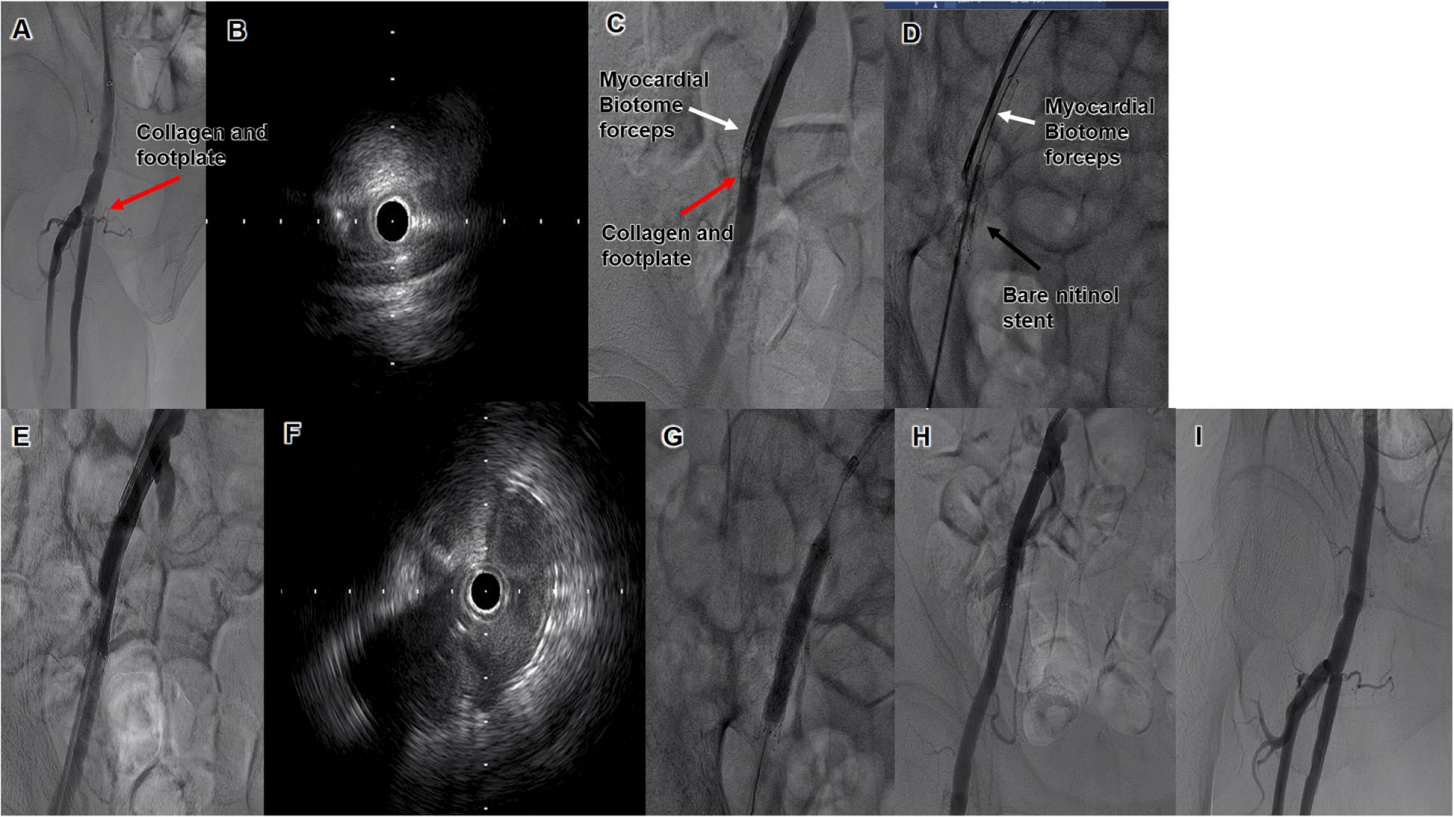
Angiogram of Case 2 after the Angio-Seal drop. **A** The Angio-Seal collagen has stopped at the bifurcation of the deep femoral artery (DFA). **B** Intravascular ultrasound (IVUS) observation from the superficial femoral artery (SFA) shows that the Angio-Seal collagen occupies the SFA and almost excludes the SFA. **C** A biotome grasps the Angio-Seal collagen and pulls it up to the external iliac artery (EIA). **D**. While the biotome forceps grasp the collagen, a 10.0- × 60-mm stent (SMART^®^; Cordis, USA) is deployed to the EIA and the Angio-Seal collagen is positioned between the stent and the vessel wall. **E** After stent deployment, the stent expansion is not completed. **F** IVUS observation of the portion of the stent where the collagen is inserted shows that the collagen is pushing the stent. **G** Post-dilation of the stent. **H-I** The final angiography shows good antegrade flow with no dissection or residual stenosis. The stent is well dilated and there is no remaining material at the bifurcation of the SFA and the DFA

An 88-year-old man with hypertension, dyslipidemia, and diabetes mellitus presented with claudication of the right lower limb. The ankle–brachial index was 0.88 on the right side. A 6-Fr guiding sheath (Parent Select 5082^®^ guiding sheath; Medikit) was inserted into the right CFA. The lesion was very hard, so IVUS-guided parallel wiring was performed [7]. A 0.014-inch guidewire (Crosslead penetration^®^ guidewire; Asahi Intec) was advanced through the intraplaque spaces and succeeded in crossing the lesion (Fig. 3C). A 5.0- × 150-mm drug-coated balloon (Lutonix^®^; BD, USA) was dilated at the popliteal artery lesion (Fig. 3D and E). We used the Angio-Seal hemostatic device (Terumo) to achieve puncture site hemostasis. However, hemostasis failed. A 6-Fr guiding sheath (Crossroads^®^ guiding sheath; NIPRO) was inserted into the left CFA via the contralateral approach. Control angiography showed that the Angio-Seal collagen was stuck at the bifurcation of the SFA and DFA (Fig. 4A). A 0.014-inch guidewire (Jupiter FC^®^ guidewire; Boston Scientific) was inserted into the SFA. IVUS (Altaview^®^ IVUS; Terumo) showed that the Angio-Seal collagen had dropped into the CFA and into the SFA and had almost occluded the SFA (Fig. 4B). MBF were inserted and used to grasp the collagen and lift it to the right EIA (Fig. 4C). Additionally, the distal CFA was punctured by angiography-guided and a 6-Fr short sheath was inserted. A 10.0- × 60-mm BNS (SMART^®^; Cordis) was deployed to the right EIA so that the MBF grasping the collagen could be positioned between the stent and the vessel wall (Fig. 4D and F). After dilation, angiography and IVUS observations showed that the stent was appropriately expanded and no material remained (Fig. 4G and H). The final angiography showed good antegrade flow (Fig. 4I). Bilateral hemostasis was performed by using Exoseal (Cordis) hemostatic devices.The time from the first angiogram to the last angiogram was 28 min. Postoperative antiplatelets regime was the same as in case 1.

## Discussion

We demonstrated that the CLAW CRANE technique was an effective bailout EVT for Angio-Seal collagen drop. In these two cases, we were able to recover the femoral artery occlusion safely and effectively by trapping the collagen with the footplate between the iliac artery vessel wall and the BNS. After confirming that the BNS had covered the collagen area, the biotome was released from the grasp of the MBF, and the MBF were withdrawn. Although the MBF were trapped by the BNS, they were easily removed due to the straight structure. If the collagen is let go before stent placement, it may be dropped distally. In both cases, this procedure was successfully performed without any problems, and the MBF were removed without any resistance.

Previous studies have reported several EVT bailout methods. One study used a balloon-mounted bare metal stent and trapped the collagen between the stent and the SFA vessel wall [8]. Our method trapped the collagen between the wall of the iliac artery and the stent, as the patency is reportedly excellent at 5 years after iliac artery stenting [9, 10]. If the collagen has fallen from the CFA to the femoral artery bifurcation, balloon dilation or implantation of a stent may obstruct the DFA and should be avoided if possible. Furthermore, the long-term patency of the BNS seems to be better after implantation in the iliac artery than implantation in the SFA [11]. However, if fragments migrate distally into the SFA, transpopliteal retrograde approach is one option for this bailout technique.

It would be ideal if all of the Angio-Seal collagen with the footplate could be removed after grasping with the MBF. There was report of successful endovascular bailout management of a dislocated Angio-Seal with use of an Alligator Tooth Retrieval forceps [12]. In Case 1, we tried to place the collagen into the 6-Fr guiding sheath after grasping it with the MBF, but it did not fit. IVUS showed that the collagen was large enough to occupy the DFA, and we would have had to insert a very large sheath to retrieve it, which would have been quite difficult from the contralateral crossover approach. Although 8-Fr guiding sheath could be inserted from the contralateral side, there was no proof that collagen with footplate could be inserted within 8-Fr. We also considered inserting a large-diameter sheath retrogradely from the distal CFA with the collagen grasped by the MBF and inserting the collagen into it, but we did not do so because of the risk of new puncture site complications.

In most cases, surgical treatment is one of the golden standard methods for managing Angio-Seal failure [13]. However, surgical treatment usually requires a general anesthetic, and patients with peripheral artery disease tend to have other comorbidities and are considered high risk for general anesthesia. In our method, the patients are treated only with EVT and therefore only require local anesthesia at the puncture site; additionally, the procedural time was around 30 min from the preoperative angiography to the final angiography.

Vascular closure device failure is reported to occur in 2.7% of patients, and once it occurs, it leads to significant increase in the risk of vascular complications [14]. We performed angiography-guided femoral artery punctures in the two cases presented here, and decided whether an Angio-Seal haemostatic device could be used based on contrast findings, fluoroscopic calcification, stenosis, and branching. DUS-guided femoral artery punctures and consideration of indications for Angio-Seal haemostatic devices might be useful in preventing complications. It may be difficult to diagnose Angio-Seal failure because the device produces consistent artifacts when scanned immediately after deployment [15]. In both cases, we performed DUS and angiography immediately after Angio-Seal failure was suspected. The cause of Angio-Seal failure seemed to be inappropriate placement of the anchor or insufficient extrusion of the anchor. As there is no perfect method to eliminate complications, it is useful for clinicians to be aware of bailout techniques such as the CLANE CRAW technique that we proposed in this study.

In the present two cases, the CLAW CRANE bailout procedure was completed in a very short time with no complications. However, the efficacy and the safety of our method is not known because the number of patients was very small. A much larger study is needed to confirm the safety and efficacy of our bailout method.

## Conclusions

The CLAW CRANE technique using MBF to grasp the dislodged collagen with the footplate and covering it with a BNS at the iliac artery may be useful for managing Angio-Seal failure.

## Data Availability

The datasets used and/or analyzed during the current study are available from the corresponding author on reasonable request.

## Abbreviations

CFA: Common femoral artery
DUS: Duplex ultrasound
MBF: Myocardial biotome forceps
BNS: Bare nitinol stent
CTO: Chronic total occlusion
IVUS: Intravascular ultrasound
EIA: External iliac artery
SFA: Superficial femoral artery
DFA: Deep femoral artery
EVT: Endovascular therapy
